# Deciphering biodiversity and interactions between bacteria and microeukaryotes within epilithic biofilms from the Loue River, France

**DOI:** 10.1038/s41598-017-04016-w

**Published:** 2017-06-28

**Authors:** Anouk Zancarini, Isidora Echenique-Subiabre, Didier Debroas, Najwa Taïb, Catherine Quiblier, Jean-François Humbert

**Affiliations:** 1 iEES Paris, UMR UPMC-CNRS-IRD-INRA-Univ. Paris, 7-UPEC Paris, France; 2 Unité Molécules de Communication et Adaptation des Microorganismes, Muséum National d’Histoire Naturelle, CNRS, Paris, France; 3 Laboratoire “Microorganismes: Génome et Environnement”, Clermont Université, Clermont-Ferrand, France; 4 Laboratoire “Microorganismes: Génome et Environnement”, CNRS, Aubière, France; 5 Université Paris Diderot, Paris, France

## Abstract

Epilithic river biofilms are complex matrix-enclosed communities harboring a great diversity of prokaryotic and eukaryotic microorganisms. Interactions between these communities and the relative impacts of environmental factors on their compositions are poorly understood. In this study, we assessed the spatio-temporal variation in the diversity and composition of bacterial and microeukaryotic communities within biofilms in a French river. Significant changes were found in the composition of these microbial communities over the sampling period and between the upstream and downstream stations. In addition, the beta diversity of the bacterial community tended to decrease along the river, mostly as a result of turnover. These changes could be caused by the different water temperatures and geological and hydrological river contexts at the sampling sites (from karst landscape to river plain). Finally, our network analysis showed multiple correlations among dominant OTUs. Among them, negative correlations between *Rhodobacteraceae* and two other dominant groups of photosynthetic microorganisms (cyanobacteria and diatoms) were particularly interesting, which raises the question of what environmental factors trigger the changes occurring in benthic microbial photosynthetic communities.

## Introduction

Epilithic river biofilms (attached to gravel and stones) are complex matrix-enclosed communities that can be described as microbial landscapes^1^. Prokaryotic and eukaryotic microorganisms are closely associated within these biofilms. The diversity of these communities and the ontogenesis of biofilms have been described in the literature. For example, bacteria, mainly belonging to Betaproteobacteria^2, 3^, and diatoms^4, 5^ have fundamental roles in substrate colonization by producing extracellular polymeric substances (EPS)^6^. These pioneer microorganisms facilitate the establishment of the next arrivals, including heterotrophic and photosynthetic microorganisms^7^, such as bacteria belonging to Alphaproteobacteria and Bacteroidetes^3^, cyanobacteria, microalgae^7–9^, and other microorganisms (*i*.*e*., archaea, fungi, protozoa, small metazoans, and viruses^1, 10, 11^).

From a functional point of view, cyanobacteria, diatoms, and green algae are recognized as principal primary producers in periphyton^12, 13^; however, other potentially photosynthetic bacterial taxa are also frequently detected in stream biofilms, including purple bacteria (*e*.*g*., refs 10, 14–16). Among them, members of the *Rhodobacter* genus are able to grow under anaerobic (phototrophic) and aerobic (chemoheterotrophic) conditions^17^. Autotrophic microorganisms have been described as the principal producers of the organic matter that is used by heterotrophic or mixotrophic microorganisms in biofilms^18, 19^, while allochthonous organic matter carried by water generally contributes only a minor portion of the carbon^20^. Finally, predators, such as protists exploiting biofilms as a food source, are the drivers of carbon transfer to higher trophic levels^21^.

In most papers addressing the microbial communities of stream biofilms, the microalgal and bacterial components have been described separately. On the one hand, the microalgal component has attracted the attention of numerous studies dealing with the use of these microorganisms as bioindicators for water quality assessments (*e*.*g*., refs 22–25) and the identification of environmental factors and processes impacting biofilm development in lotic environments^26^. On the other hand, numerous studies have been performed on the composition of bacterial communities in periphytic biofilms using 16S rRNA fingerprinting methods and, more recently, high-throughput sequencing approaches^27^, with the goal to better understand the spatial and temporal variation occurring in these communities^28–30^.

To our knowledge, only two works have simultaneously addressed both components (prokaryotes and eukaryotes) using high-throughput sequencing: Levi *et al*.^31^ compared the composition of epipsammic and epiphytic biofilm communities across habitats with varying physical substrates and environmental conditions, and Bricheux *et al*.^15^ characterized the microbial diversity of biofilms by testing different sets of primers on biofilms growing on glass substrates at one site in a French river. Consequently, there are no data based on the use of high-throughput sequencing that simultaneously describe the spatio-temporal variation in the composition of bacterial and microeukaryotic communities in epilithic stream biofilms despite the possible application of such approaches for identifying the relative impacts of environmental factors on these two communities and the putative interactions between them. To address this paucity of data, we performed a study on epilithic biofilms collected during summer 2012 at four sampling sites in the Loue River, located in the eastern part of France. Physicochemical parameters were recorded with the aim to better understand the variation in microbial communities. The beta diversity was analyzed to identify the relative importance of two biological processes (species replacement and species loss) involved in the variation in microbial community composition. In addition to the characterization of prokaryote and microeukaryote communities using high-throughput sequencing of 16S and 18S rRNA gene fragments, the biofilm biomass was quantified and the photosynthetic microorganisms (*i*.*e*., diatoms, cyanobacteria, and green algae) were microscopically examined to validate some of the findings provided by our molecular tools. Finally, a network analysis was performed to obtain an overview of the positive and negative relationships between the dominant bacterial and microeukaryote OTUs within biofilms and some environmental variables.

## Results

### Spatio-temporal changes in environmental conditions, biofilm biomass, and the composition of photosynthetic communities

**Figure 1:**
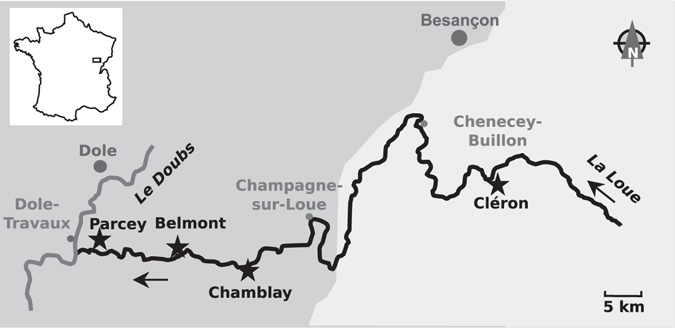
Geographic locations of the sampling sites (represented by stars) in the Loue River. Arrows represent flow direction. Lighter zones represent altitudes more than 300 meters above sea level. This figure was drawn by the authors using Inkscape 0.91 (www.inkscape.org).

At the different sampling stations along the Loue River (Fig. 1), four major high-precipitation events followed by episodes of high flow rates in the river were recorded (05/20, 06/07, 07/01, and 08/24; see Supplemental Fig. 1A,B). At the same time, increases in suspended matter, turbidity, and phosphate concentration were also detected at the Chenecey-Buillon station (Supplemental Fig. 1C,D). Only one period of low and stable water flow was observed, between mid-July and the end of August. While pH was relatively stable among the different sampling sites and dates, spatial and temporal variability in water temperature was observed (Table 1). The water temperature was lower at Cléron than at the other sites and higher in August than in June, July and September, as expected at this latitude.

**Table 1:** Environmental and physico-chemical data measured at each sampling site and date.

Sampling site	Sampling date	Water depth (cm)	Water pH	Water temperature (°C)	Current velocity (m.s.^−1^)
Cléron	June	NA	8.7	11.7	0.1 ± 0.2
	July	NA	8.6	11.6	0.7 ± 0.1
	August	29 ± 6	8.6	15.3	0.3 ± 0.2
	September	32 ± 3	8.7	13.3	0.5 ± 0.3
Chamblay	July	NA	8.5	17.1	0.2
	August	46 ± 5	8.4	23.0	0.3 ± 0.0
	September	19 ± 12	8.4	18.2	0.3 ± 0.1
Belmont	July	43 ± 3	8.5	17.3	0.6 ± 0.2
	August	46 ± 12	8.3	22.1	0.3 ± 0.1
	September	42 ± 9	8.4	18.6	0.5 ± 0.3
Parcey	June	NA	8.8	15.5	0.7 ± 0.5
	July	22 ± 4	8.5	16.3	0.3 ± 0.1
	August	25 ± 1	8.3	20.6	0.3 ± 0.1
	September	34 ± 15	8.5	17.2	0.6 ± 0.2

Values (± s.d.) corresponded to means of three replicate environmental measurements.

**Figure 2:**
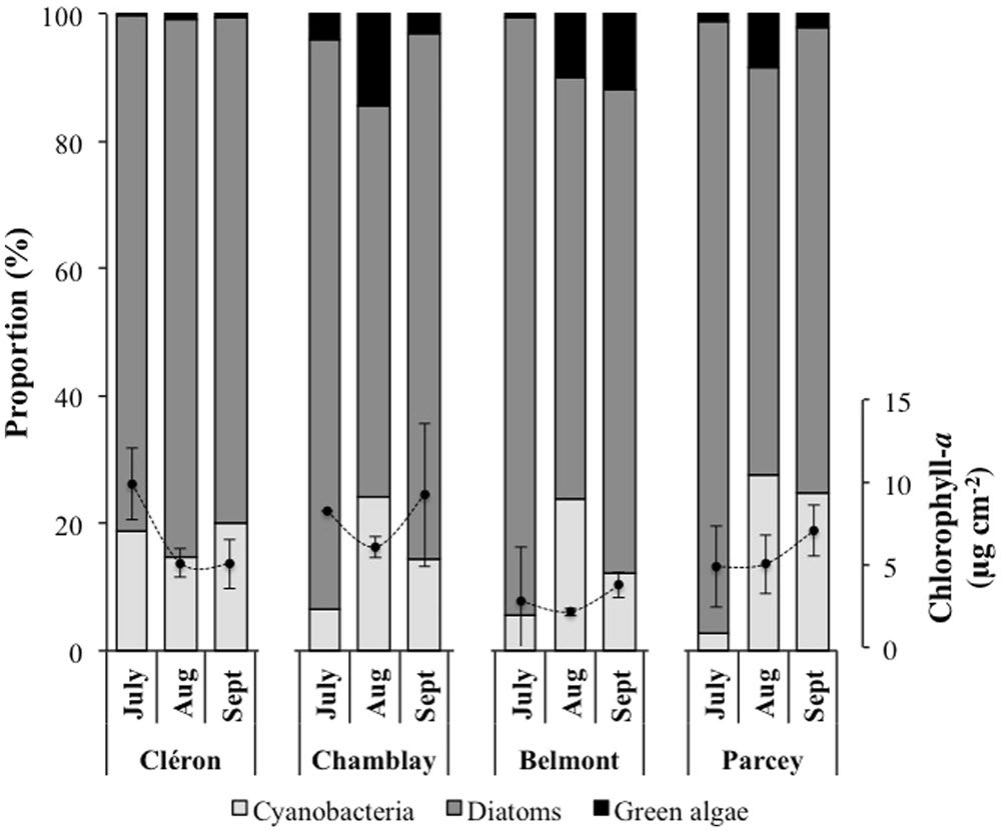
Spatio-temporal variation in the proportions of the main photosynthetic taxonomic groups in the Loue River biofilms estimated by microscopic cell counting (stacked histogram) and in chlorophyll-*a* concentrations (black circles). Values and error bars represent the means of the three replicates and the standard deviations, respectively, except for Chamblay in July, where only one sample was collected due to poor weather conditions. Abbreviations: Aug, August; Sept, September.

Total chlorophyll-*a* (Chl-*a*) concentrations ranged between 2 and 10 μg cm^−2^ (Fig. 2). Higher values were found at the end of the sampling period (September) except at Cléron (the most upstream station), where the Chl-*a* concentrations were higher in July. In addition, the biofilms collected from Belmont showed significantly lower biomasses compared to those from Cléron and Chamblay (2-way ANOVA; *P* < 0.01; Supplemental Table 1).

Regardless of the sampling site and date, diatoms always dominated the photosynthetic communities, followed by cyanobacteria and then green algae (Fig. 2). In contrast to diatoms, the proportion of cyanobacteria increased significantly in August/September at three of the four sampling sites (Chamblay, Belmont, and Parcey) (2-way ANOVA; *P* < 0.001; Supplemental Table 1), and there was also an increase in the proportion of green algae in August (2-way ANOVA; *P* < 0.05; Supplemental Table 1).

### Molecular characterization of the microbial communities in biofilms

#### Alpha and beta diversity of bacterial and microeukaryotic communities

The rarefaction curves and Chao1 index showed that a lower sequencing depth was required to capture the microeukaryotic diversity than the bacterial diversity (Supplemental Figs 2 and 3). Nevertheless, our data described an important fraction of the bacterial community. Interestingly no significant correlations were found in the spatial variation in the alpha diversity of the bacterial and microeukaryotic communities when comparing all sampling sites (Pearson correlation; r = 0.11, 0.23 and 0.01 for OTU number, Chao1 and Pielou’s evenness, respectively). In terms of the spatio-temporal variation in alpha diversity (Supplemental Fig. 3), our analyses revealed that (i) the evenness of the microeukaryotic communities was higher in June and July than in August and September (2-way ANOVA; *P* < 0.05) and (ii) the richness and evenness of the bacterial communities increased from the upstream site (Cléron) to downstream sites (Belmont and Parcey) (2-way ANOVA; *P* < 0.05), while the opposite was found for evenness in the microeukaryotic communities.

**Figure 3:**
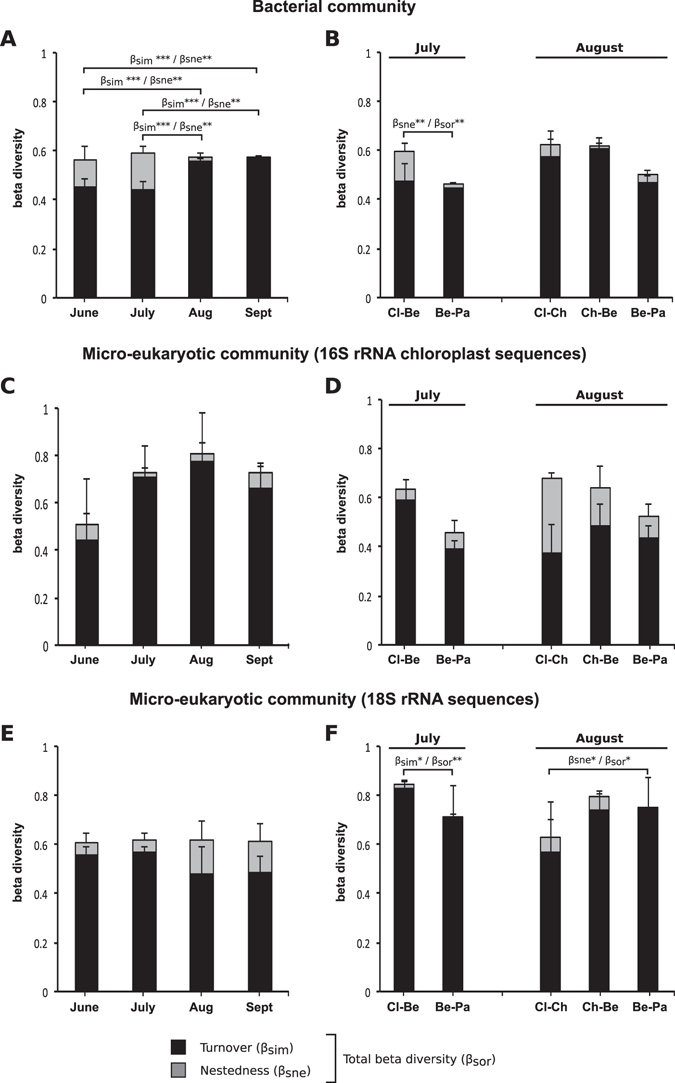
Temporal variation in the beta diversity of the bacterial (**A**, 16S rRNA gene) and microeukaryotic communities (**C**, 16S rRNA chloroplast sequences; **E**, 18S rRNA gene) between the Cléron and Parcey sampling stations and the spatial variation in the beta diversity in the bacterial (**B**, 16S rRNA gene) and microeukaryotic communities (**D**, 16S rRNA chloroplast sequences; **F**, 18S rRNA gene) in July and August. Histograms represent total beta diversity (β_sor_), turnover (β_sim_) = black, and nestedness (β_sne_) = gray. Error bars represent the standard deviation. Only statistically significant differences are noted: *<0.05, **<0.01, ***<0.001. Abbreviations: Aug, August; Sept, September; Cl, Cléron; Ch, Chamblay; Be, Belmont; Pa, Parcey.

Following rarefaction, 8,643 and 838 sequences per sample were used for the 16S and 18S rRNA gene analyses, respectively. When looking at the temporal variation in beta diversity in the bacterial and microeukaryotic communities, our analyses revealed that (i) the total beta diversity values (Sorensen pairwise dissimilarity) were mostly higher than 0.5 without significant differences (ANOVA; *P* > 0.05) among the different sampling dates, and (ii) most of the temporal changes in beta diversity were due to turnover (OTU replacement) (Fig. 3A,C and E). In addition, no significant difference was found in temporal variation in terms of both turnover and nestedness, except in bacterial communities, where a significant increase in the contribution of turnover was detected in August and September (ANOVA; df = 3, F = 9.56, *P* = 0.005) (Fig. 3A). Finally, regarding the sampling sites (Fig. 3B,D and F), the total beta diversity showed decreasing trends from upstream to downstream in July and August, except for the microeukaryotic communities (based on the 18S rRNA dataset) in August.

#### Composition of bacterial and microeukaryotic communities

**Figure 4:**
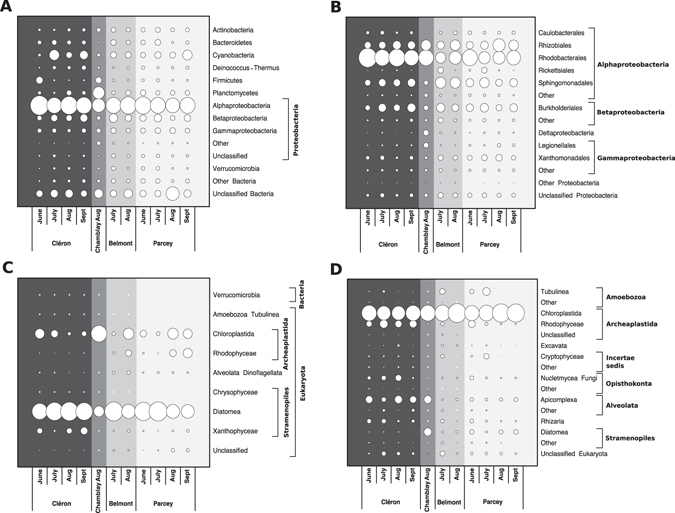
Spatio-temporal variation in the composition of the bacterial (**A**), proteobacterial (**B**), chloroplast (**C**), and microeukaryotic (**D**) communities based on 16S (**A**–**C**) and 18S (**D**) rRNA gene sequences. Circles represent the mean proportions of microbial sequences in the three replicates for each sampling site and date. Chloroplast 16S rRNA gene sequences were classified by BLAST on NCBI. Abbreviations: Aug, August; Sept, September.

Our data revealed that Proteobacteria (mean 60% ± 10) dominated the bacterial communities, followed by Cyanobacteria (mean 8% ± 6) and Planctomycetes (mean 6% ± 8) (Fig. 4A, Supplemental Table 2). Among the Proteobacteria, the order Rhodobacterales (Alphaproteobacteria) contained more than 42% of the sequences (Fig. 4B, Supplemental Table 2). When looking at the relative abundance of the bacterial OTUs, 20% of the sequences belonged to the eleven most abundant OTUs (>1% of all sequences for all the samples), and seven of these eleven OTUs belonged to the order Rhodobacterales (Supplemental Table 2). The rest of the abundant OTUs were represented by Cyanobacteria (*Chamaesiphon* sp.), and three others were unclassified.

From the sequencing of the 18S rRNA, it appeared that Chloroplastida (mean 68% ± 16), entirely represented by Chlorophyta, were dominant in the microeukaryotic community of the Loue River biofilms, followed by Alveolata (mean 8% ± 8, principally represented by Apicomplexa), Rhodophyceae (mean 7% ± 8), Stramenopiles (mean 4% ± 5), Tubulinea (mean 3% ± 5), Fungi (mean 3% ± 5), Rhizaria (mean 2% ± 3) and Cryptophyceae (mean 1% ± 3) (Fig. 4D, Supplemental Table 3). Moreover, 14 OTUs contained 78% of all the sequences, with seven of them belonging to the Chlorophyta division, while the rest were affiliated with Rhodophyceae, Tubulinea, Alveolata and Stramenopiles (Supplemental Table 3).

The composition of the microeukaryotic community was also characterized using a BLAST analysis, which was performed on the 26,124 16S rRNA sequences affiliated with chloroplasts using the PANAM pipeline. In contrast to the results obtained for the 18S rRNA sequences, 92% of the chloroplast sequences were affiliated with Diatomea, while only 4% were affiliated with Chloroplastida (Fig. 4C, Supplemental Table 4). Diatomea represented 17 of the 19 most abundant OTUs, while the rest of the most abundant OTUs were affiliated with Chlorophyta and Dinoflagellata (Supplemental Table 4).

Consequently, these two analyses performed on the microeukaryotic communities provided contrasting findings on the relative proportions of diatoms and green algae in our samples. However, the microscopic enumerations highlighted a large dominance of diatoms, in agreement with the chloroplast sequences.

### Spatio-temporal changes in the bacterial and microeukaryotic biofilm communities

**Figure 5:**
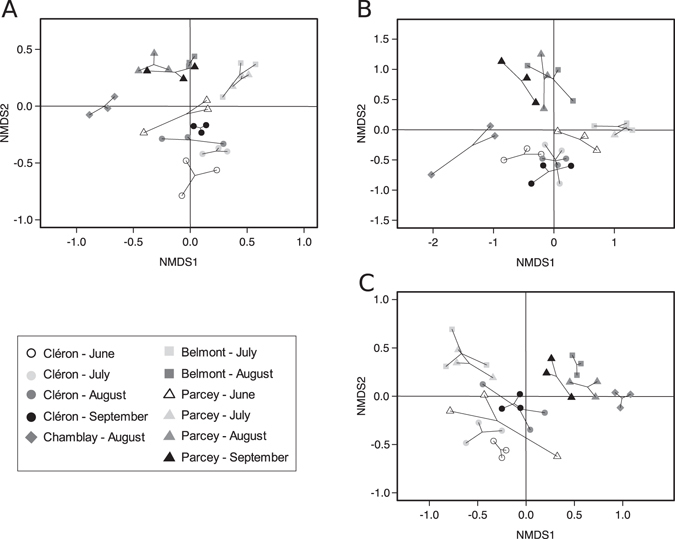
Nonmetric multidimensional scaling (NMDS) representing the diversity of the bacterial (**A**) and microeukaryotic communities (**B**,**C**) among the different sampling sites and dates. NMDS was performed using the 16S rRNA pyrosequencing dataset without chloroplast sequences (**A**), the 16S rRNA chloroplast pyrosequencing dataset (**B**), and the 18S rRNA pyrosequencing dataset (**C**).

Nonmetric multidimensional scaling (NMDS) analyses were performed on the distribution of all OTUs for both the bacterial and microeukaryotic communities (based on the rarefied datasets of 16S rRNA without chloroplast sequences and 16S rRNA chloroplast and 18S rRNA sequences, respectively) (Fig. 5). Significant effects of sampling site, sampling date and the interaction between sites and dates (PERMANOVA; *P* < 0.001, Supplemental Table 5) were detected. First, a clear distinction was found between the microbial communities sampled in June/July and those sampled in August/September in all three NMDS analyses (Fig. 5) except for those from the upstream sampling station of Cléron (Fig. 5A and B, respectively). When evaluating the composition of these communities in more detail (Supplemental Table 6), significant increases in Rhizobiales and Chloroplastida (particularly members of Chlorophyceae; based on the 18S rRNA dataset) were found in August/September compared to July, while numerous bacteria belonging, for example, to the phyla Acidobacteria, Bacteroidetes and Nitrospirae (based on the 16S rRNA dataset) and microeukaryotes such as Ulvophyceae (based on the 18S rRNA dataset) and Diatomea (based on the 16S rRNA chloroplast dataset) displayed significant decreases in August/September (ANOVA and Tukey’s test; Supplemental Table 6).

Second, the bacterial and microeukaryotic communities displayed spatial differentiation, particularly between Cléron and the other sampling sites (Fig. 5). When evaluating the community composition in more detail (Supplemental Table 6), significant differences were found between Cléron and the other sampling sites in terms of the relative abundance of some microbial groups, such as Rhodobacteraceae and Rhodophyceae, which were overrepresented in Cléron; conversely, Chlorophyceae (based on the 18S rRNA dataset), Stramenopiles (based on the 16S rRNA chloroplast dataset) and Acidobacteria were significantly underrepresented at this station (ANOVA and Tukey’s test; Supplemental Table 6). While the NMDS based on 16S rRNA chloroplast sequences better reflected the microscopic observations, the NMDS based on 18S rRNA sequences was also important in showing the spatio-temporal differences in microbial communities for Archaeplastida (Chlorophyceae, Ulvophyceae and Rhodophyceae), nonphotosynthetic microeukaryotes (Apicomplexa and Amoeba) and Cryptomonadales. Thus, these three complementary analyses showed similar spatio-temporal differences within samples. Indeed, the co-inertia analyses performed among the bacterial 16S rRNA, chloroplast 16S rRNA and microeukaryotic 18S rRNA data revealed significant pairwise correlations between all these datasets (Mantel tests; r = 0.68, 0.44, and 0.36 for comparisons between 16S bacterial/16S chloroplast data, 16S bacterial/18S microeukaryotic data, and 16S chloroplast/18S microeukaryotic data, respectively; *P* < 0.001 for the three Mantel tests).

### Co-occurrence analysis among the dominant OTUs

**Figure 6:**
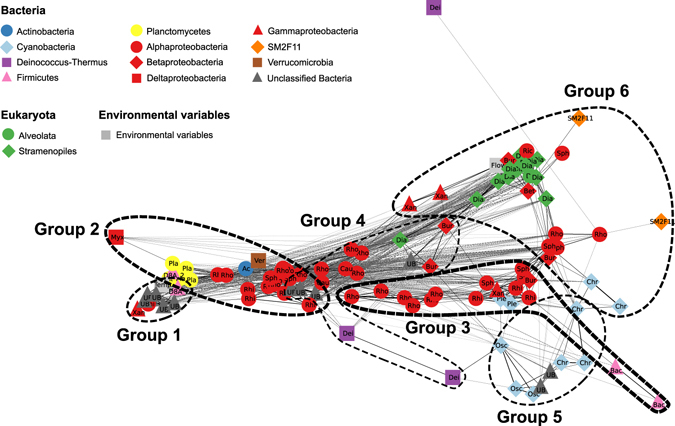
Network based on Spearman correlations among the relative abundances of the dominant bacterial and microeukaryotic OTUs and environmental data (water temperature at each sampling station and river water flow rate) for all the sampled biofilms. Only significant positive and negative correlations are represented (Spearman’s ρ > |0.5|). Nodes correspond to both microbial OTUs and environmental variables. Gray dashed and black solid lines represent negative and positive correlations, respectively. Line thickness is proportional to the value of correlations between two nodes; thick and thin lines correspond to high (near |1|) and low (near |0.5|) correlations, respectively. The six groups were defined using hierarchical clustering based on Spearman correlations generated using the hclust complete linkage method in R software. Abbreviations: Ac, Acidimicrobiales; Chr, Chroococcales; Osc, Oscillatoriales; Ple, Pleurocapsales; Dei, Deinococcales; Bac, Bacillales; Pla, Planctomycetales; Cau, Caulobacterales; Rhi, Rhizobiales; Rho, Rhodobacterales; Ric, Rickettsiales; Sph, Sphingomonadales; Bur, Burkholderiales; Myx, Myxococcales; Xan, Xanthomonadales; Ver, Verrucomicrobiales; UB, unclassified bacteria; Dino, Dinoflagellata; Dia, Diatomea; Flow, river water flow rate; Temp, water temperature at sampling station.

To assess correlations among environmental conditions and the abundance of dominant bacterial and microeukaryote OTUs within biofilms across the different sampling sites and dates, a network analysis (based on Spearman correlations using the SparCC method) was performed. Among the 110 dominant bacterial and microeukaryotic OTUs (based on 16S rRNA bacterial and chloroplast sequences), 104 displayed 861 positive and negative correlations, resulting in a complex network (Fig. 6). Among these 104 dominant OTUs, more than 44% were potentially photosynthetic microorganisms (14, 10, and 22 OTUs were identified as Diatomea, Cyanobacteria, and Proteobacteria belonging to the Rhodobacteraceae family, respectively). When looking at all the significant correlations between OTUs in the network, more positive (59%) than negative correlations were observed. Among the positive interactions, six main groups of OTUs were identified in the network and were largely dominated by one taxonomic group of microorganisms (Supplemental Fig. 4). Most negative correlations occurred between OTUs belonging to phylogenetically unrelated classes, such as those found between Diatomea and some Alphaproteobacteria OTUs (Rhodobacterales and Rhizobiales). Interestingly, cyanobacteria displayed strong positive interactions with OTUs belonging to the orders Burkholderiales, Sphingomonadales, and Rhizobiales and negative interactions with OTUs belonging to the order Rhodobacterales (Supplemental Table 7).

Finally, when considering environmental variables, river water flow rate and local water temperature displayed significant correlations with some microbial OTUs (Fig. 6). Specifically, a positive correlation was found between the global flow rate and the presence of diatoms.

## Discussion

Deciphering the composition of microbial communities in river biofilms and their interactions and identifying the impacts of environmental factors on these communities are of particular importance for attaining a better understanding of the functioning of riverine biofilms. It is essential to consider bacterial and microeukaryotic communities together because they are closely functionally associated in organic matter-producing and -recycling processes. In this framework, our study on Loue River biofilms provides new insights into the relationships between prokaryote and microeukaryotic communities.

Concerning the alpha diversity of the microbial communities in the Loue River biofilms, it appeared that there was no correlation in the spatio-temporal variation of the different alpha diversity indices between the bacterial and microeukaryotic communities. This finding suggests that there is either no direct link in the variation of alpha diversity between the bacterial and microeukaryotic communities or that there are time lags in their respective variation. The beta diversity was analyzed to identify the spatio-temporal variations occurring in the composition of microbial communities in the Loue River and the relative importance of species replacement and species loss in such variation. We found that the bacterial communities displayed a decreasing trend in beta diversity from the upstream part to the downstream part of the river, which is in agreement with previous findings by Besemer *et al*.^32^. Two main hypotheses could explain this pattern: (i) a decline in water quality from upstream to downstream, which is a common feature of many rivers (*e*.*g*., Vannote *et al*.^33^), and (ii) differences in the heterogeneity of the local environmental conditions between the upstream and downstream parts of the river, knowing that the downstream part has been rectified. Finally, the high dominance of turnover in the changes occurring in the composition of the microbial communities suggests that environmental sorting or spatial and historical constraints^34^, rather than colonization or extinction processes^35^, are likely important drivers of the composition of these microbial communities.

Among the changes occurring in both communities (bacteria and microeukaryotes), it is interesting to consider the variation in the proportions of cyanobacteria, diatoms, and green algae. With the exception of the sampling station at Cléron, the increase in water temperature associated with a decrease in water velocity in August and September led to concomitant increases in the proportion of cyanobacteria and green algae and a decrease in the proportion of diatoms at the downstream stations. These findings are in agreement with other studies describing diatoms as “cool season species” and cyanobacteria as “warm/hot season species”^36–38^. We found that microbial communities from the upstream station (Cléron) displayed fewer changes in the seasonal variation of their composition compared to those sampled from the downstream stations. These observations are interesting when looking at the characteristics of the river. In the Loue River, the upstream part is located in a karst landscape, while the downstream part is located in a river plain^39, 40^. Moreover, in the upstream part of this river, the water temperature was 5 °C lower on average than in the downstream part, and the water temperature at the Cléron station displayed less temporal variation compared to the other stations due to multiple cold water resurgences occurring along this part of the river. Altogether, these findings support the essential role of water temperature in the variation of the composition of all biofilm microbial communities, not only on diatoms and cyanobacteria. These findings also emphasized the necessity of taking the geological and hydrological contexts of the sampling stations into account when working along such a river.

One other striking point of this study concerns the high number of reads assigned to bacteria other than cyanobacteria that are potentially able to contribute to primary production in these river biofilms. Indeed, 26% of the bacterial reads correspond to potentially photosynthetic microorganisms belonging to the Rhodobacteraceae family, confirming previous studies highlighting the large abundance of purple non-sulfur bacteria (*e*.*g*., refs 10, 14–16). Even if most of these bacteria can also be considered heterotrophic^17^, with their photoautotrophic metabolisms depending on environmental conditions (anaerobic conditions, for example), this finding suggests that it might be interesting to estimate their contribution to primary production in river biofilms by using, for instance, an isotope labeling approach.

Finally, concerning the multiple interactions occurring between microorganisms in river biofilms, it appeared that the strongest positive correlations occurred between OTUs belonging to the same taxonomic group (for example, between diatoms). This suggests that the environmental conditions leading to the high abundance of a given group combined, for example, with microenvironmental heterogeneity within the biofilms or with predation or parasitism, allow the promotion and maintenance of a high level of richness within the group, even if one species can be temporally dominant. This observation is interesting to consider knowing that species that are phylogenetically closely related generally display high functional redundancy (*e*.*g*., Martiny *et al*.^41^) and that this functional redundancy supports biodiversity and ecosystem function (*e*.*g*., Wohl *et al*.^42^). When more carefully considering the interactions between photosynthetic microorganisms (cyanobacteria and microalgae) and bacteria, an interesting result concerns the fact that cyanobacterial and diatom OTUs displayed negative correlations with Rhodobacterales OTUs but positive correlations with OTUs belonging to Burkholderiales and Sphingomonadales. Bacteria belonging to these two orders are known for their high capacity to degrade complex organic matter (*e*.*g*., refs 43 and 44). Their high abundance in biofilms dominated by diatoms or cyanobacteria suggest that they might play major roles in the degradation of organic matter produced by these benthic photosynthetic microorganisms. By contrast, the negative correlations between Rhodobacteraceae and Cyanobacteria/diatoms might reflect competition between these photosynthetic microorganisms or the impact of environmental conditions on their relative importance, considering that Rhodobacteraceae need anaerobic conditions for their photosynthetic activity, while Cyanobacteria need aerobic conditions.

From a more technical point of view, we identified two main issues in terms of our molecular approach. The first issue concerns the contrasting results obtained for the structure and composition of the microeukaryotic communities when using 454 pyrosequencing of the 18S rRNA gene and microscopic cell counting. The over-representation of Chlorophyta in the 18S rRNA sequences could potentially be explained by the number of copies of the rRNA operon in the cells. Indeed, while the number of 16S rRNA gene copies per cell varies from one to fifteen among bacteria^45, 46^, the number of 18S rRNA gene copies per cell varies from one to hundreds of thousands among eukaryotes^47–49^. Furthermore, Zhu *et al*.^48^ and Godhe *et al*.^50^ highlighted a significant relationship between cell biovolume and the number of rRNA gene copies per cell in marine phytoplanktonic communities. However, the assessment of the structural diversity of the microeukaryotic communities using the chloroplast 16S rRNA gene was much more congruent with our microscopic observations. Using a cloning-sequencing approach, Shi *et al*.^51^ found that when evaluating the diversity of picoeukaryotic communities in marine environments, studies using the 18S rRNA gene clone libraries were heavily biased toward heterotrophic cells, while *psbA* or specific 18S rRNA gene primers could be more effective for targeting plastid gene clone libraries, such as 16S rRNA. The second technical issue concerns the high number of “unclassified bacteria” (843 OTUs, representing from 5 to 34% of the total bacterial community) detected among the samples. In our study, taxonomic assignment of all OTUs was performed using the LCA method. In contrast to the LCA method, the nearest neighbor (NN) method never returns “unclassified bacteria”. However, Taib *et al*.^52^ demonstrated that LCA provides more accurate assignment than the NN method, irrespective of the taxonomic level considered (from kingdom to genus).

In conclusion, this study revealed several important findings concerning the structural diversity of river biofilms and the putative interactions occurring between the numerous OTUs existing within them. Among the main questions, it would be interesting to better understand the relative contributions of all potentially photosynthetic microorganisms in the primary production of these river biofilms, considering the high abundance of some groups, such as Rhodobacteraceae, that have not previously been considered major contributors to primary production in these ecosystems. One important finding of this work is that the different hydrological and geological contexts of the Loue River have indirect impacts on the seasonal variation in the composition of microbial communities through their direct impacts on water temperature. Finally, this work also allowed us to emphasize that data provided using molecular approaches must be considered with caution and validated when possible with other approaches, such as microscopic examination.

## Methods

### Sites and sample collection

Samples were collected during summer 2012 (5–6 June, 9–10 July, 13–14 August and 10–11 September) from four sites located along the Loue River, which belongs to the Loue River system in France (for a description of this system, see Verneaux *et al*.^53^) (Fig. 1). At each site, samples were taken at three points along a transect parallel to the water’s edge and positioned one to two meters from the shoreline. All cobbles (4–15 cm length) contained within a known area defined by the surface of an underwater viewer (707 cm^2^) were collected at each point. The cobbles were then scrubbed, and the biomass was collected from 250 mL of the river water. Aliquots (5 mL) were filtered for Chl-*a* (GF/C Whatman; n = 36, no samples in June) and DNA extraction (polycarbonate 0.2 μm GTTP Millipore; n = 39, no sample from Chamblay in July due to poor weather conditions), stored chilled in the dark, and subsequently frozen (−20 °C) for later analysis in the laboratory. Subsamples (1 mL; n = 36, no samples in June) were fixed with Lugol’s solution and stored at 4 °C for later microscopic identification and enumeration.

### Environmental and physico-chemical parameters

The local water temperature, pH, current velocity and depth were measured at each sampling point on each sampling date. In addition, the flow rate was obtained from three survey stations (Chenecey-Buillon, Champagne-sur-Loue and Parcey; Fig. 1) from Hydro France (http://hydro.eaufrance.fr). Precipitation amounts were acquired for the Chenecey-Buillon and Dole-Tavaux stations (Fig. 1) from Infoclimat (http://infoclimat.fr). Moreover, phosphates (PO_4_), dissolved oxygen (DO), and suspended matter concentrations, along with conductivity, pH, and turbidity, were obtained for the Chenecey-Buillon station from the Port Douvot depuration station.

### Chlorophyll-a extraction and quantification

Glass microfiber filters containing samples were extracted in darkness with 90% methanol (10 mL) in Falcon tubes (15 mL) according to a protocol described in Echenique-Subiabre *et al*.^54^. The equation of Talling and Driver^55^ was used to estimate the chlorophyll-a (Chl-a) concentration (µg L^−1^), which was then transformed to surface unit concentration (mg cm^−2^) using the filtered biomass volume value and the sampling area defined by the surface of an underwater viewer (707 cm^2^).

### Counting procedure and identification of photosynthetic microorganisms

Lugol’s iodine-preserved samples were homogenized and diluted in Milli-Q water. Identification and enumeration of the samples were performed as described previously^54^. Cells were only identified as belonging to cyanobacteria, diatoms or green algae. Cells from other taxonomic groups were not considered, as they were always in the ultra-minority.

### DNA extraction

The DNA extraction procedure was based on mechanical and chemical extraction as described previously in Zhu *et al*.^56^ for each sample (n = 39) on one-quarter of each polycarbonate filter.

### PCR and pyrosequencing

The V4–V5 region of the 16S rRNA gene (16S rDNA) was selected for tag pyrosequencing. This region was amplified using the bacterial forward primer 563F^57^, which also included the Roche 454 pyrosequencing adapter FLX A and a unique 10-bp barcode. The bacterial reverse primer 907rM^58^ was also used and included the Roche 454 pyrosequencing adapter FLX B Polymerase chain reaction (PCR) was performed as described previously^56^.

For 18S rRNA gene sequencing, the V1–V2 region was amplified using the forward primer P45F^59^, which also included the Roche 454 pyrosequencing adapter FLX A and a unique 10-bp barcode, and the reverse primer P47R^59^, which included the Roche 454 pyrosequencing adapter FLX B adaptor. The PCR volume of 50 µL contained 1X Phusion HF Buffer, 1 mM MgCl_2_, 0.2 mM each deoxynucleotide, 0.4 μM each primer, 1 U Phusion HF Polymerase (Thermo Scientific, EU Lithuania), and 10 ng of DNA template and was completed with up to 50 µL nuclease-free water. PCRs were performed under the following conditions: 98 °C for 1 min; 30 cycles of 98 °C for 10Sec, 57 °C for 30Sec and 72 °C for 50Sec; and 72 °C for 10 min.

Each DNA extract was amplified using three replicate PCRs, which were then pooled. These PCR products were then purified using a MinElute PCR purification kit (Qiagen, Venlo, the Netherlands). The amount of DNA in each sample was quantified using the Qubit dsDNA HS assay (Invitrogen, Carlsbad, CA, USA). Finally, the PCR products (n = 33, no sample from Chamblay in July due to poor weather conditions and no samples from Chamblay and Belmont in September due to the absence of PCR amplification) were combined together in equimolar amounts and sequenced using a GS FLX Titanium 454 Genome Sequencer (GATC Biotech, Roche Company, Branford, CT, USA).

### Bioinformatics analysis

The 454 pyrosequencing of the 16S and 18S rRNA genes produced 644,701 and 392,109 raw sequences, respectively. All sequences were cleaned by applying PANGEA trimming^60^ with a quality threshold >23, minimum sequence lengths of 270 bp for 16S rRNA and 200 bp for 18S rRNA genes, and removal of sequences with errors in the forward primer and chimeric sequences using UCHIME^61^ (representing 11.4% and 2.2% of the sequences for 16S rRNA and 18S rRNA genes, respectively). The remaining sequences were clustered into operational taxonomic units (OTUs) using USEARCH^62^ at 97% and 95% similarity thresholds for bacterial and microeukaryotic sequences, respectively^63^. All OTUs were initially assigned to phyla using the SILVA database^64^, and then phylogenies containing both reference and query sequences were built *de novo*. The resulting phylogenies were then parsed to assess the taxonomy of the query sequences according to their neighboring reference sequences. The process was automated using PANAM (https://github.com/panammeb/), which constructs phylogenetic trees for taxonomic annotation^52^. The assignment method used was LCA (lowest common ancestor). After the removal of singleton and bacterial sequences identified as chloroplasts (for 16S rRNA gene analysis), a range of 8,644 to 24,960 sequences was obtained for the bacterial communities, and a range of 839 to 16,006 sequences was obtained for the microeukaryotic communities. For the assessment of the beta diversity of the bacterial and microeukaryotic communities and the description of their composition and spatio-temporal variation, the 16S and 18S rRNA gene sequence datasets were rarefied to equal sample sizes based on the sample with the fewest sequences (8,643 and 838 sequences, respectively) using a random subsampling procedure programed in the statistical software package R 3.1.0 (R Development Core Team, Vienna, Austria^65^; personal R script). For the bacterial communities, 7,478 OTUs were obtained from the 285,219 sequences analyzed (8,643 sequences per sample). For the microeukaryotic communities, 456 OTUs were obtained from the 27,654 sequences analyzed (838 sequences per sample). The microeukaryotic community was also characterized by the analysis of the 26,124 16S rRNA gene sequences affiliated with chloroplasts (SILVA database), which were then classified by BLAST on NCBI. OTUs were classified as abundant^66^ (≥1%), intermediately abundant (<1% and >0.01%), and rare (≤0.01%)^67^. Eukaryotic and cyanobacterial classifications were updated according to the latest revised classifications from Adl *et al*.^68^ and Castenholz *et al*.^69^, respectively.

### Statistical analysis

All statistical analyses were performed and figures were constructed using the statistical software package R 3.1.0^65^. Only significant differences at *P* < 0.05 were considered.

Sampling site and date effects on biomass (Chl-*a*, cyanobacteria, diatoms, and green algae), diversity indices, and the abundance of microbial groups were analyzed using 2-way ANOVA tests followed by Tukey’s test.

Alpha diversity indices (richness, Chao1 and Pielou’s evenness) and rarefaction curves were calculated using the package vegan^70^. Richness represents the OTU number (at 97% and 95% similarity thresholds for bacterial and microeukaryotic sequences, respectively), Chao1 estimates the total richness, and Pielou’s evenness provides information about the equity in OTU abundance. Correlation coefficients between bacterial and microeukaryotic diversity indices were calculated with the “cor” function using Pearson’s method. The beta diversity was also estimated; conceptually, beta diversity is the extent of change in the community composition^71^ among sites within a geographical area of interest. In this study, we evaluated the beta diversity at the OTU level to measure the variation among sites and dates for the bacterial (16S rRNA without chloroplast sequences) and microeukaryotic (assessed from 16S rRNA chloroplast and 18S rRNA sequences) communities separately. Following the procedure described by Baselga^34^, beta diversity was estimated based on presence-absence data using pairwise Sorensen dissimilarities to compare the spatial and temporal variations. On each date and for each dataset (16S rRNA without chloroplast sequences, 16S rRNA chloroplast sequences and 18S rRNA sequences), beta diversity was estimated between the most upstream site (Cleron) and the most downstream site (Parcey) by pairwise Sorensen comparison from June to August. Beta diversity was also estimated site by site from upstream to downstream (pairwise Sorensen comparison) in July and August. For each analysis, we obtained the total beta diversity (β_sor_) and its partitioning in terms of turnover (OTU replacement; β_sim_) and nestedness (OTU loss or gain; β_sne_) using the betapart package^72^.

NMDS analyses were performed on the rarefied 454 pyrosequencing datasets (bacterial sequences without sequences identified as chloroplasts, microeukaryotic sequences and bacterial sequences identified as chloroplasts) using the package vegan^70^. Three Hellinger transformations were first performed on the rarefied 454 pyrosequencing datasets; subsequently, principal component analyses (PCAs) and co-inertia analyses were performed using the package ade4TkGUI^73^. Permutational multivariate ANOVA (PERMANOVA) and Mantel tests were performed to test for differences in the bacterial and microeukaryotic (16S rRNA chloroplasts and 18S rRNA) communities among the different sampling sites and dates using the vegan package^70^.

### Network analysis

To identify associations among the dominant bacterial and microeukaryotic OTUs (≥ 1% of the total sequences for at least one of the 33 samples) and environmental data (water pH, water temperature, current velocity at sampling sites and Loue River flow), we calculated Spearman correlations using the SparCC method (Sparse Correlations for Compositional data^74^; available at https://bitbucket.org/yonatanf/sparcc) among the relative abundance levels of dominant bacterial and microeukaryotic OTUs (based on the 454 pyrosequencing dataset of the 16S bacterial and chloroplast rRNA gene sequences) and environmental data for all the samples (n = 33). The resulting matrix of correlation coefficients was parsed in R software using the function “exportNetworkToCytoscape” in the WGCNA library. Only correlations <−0.5 and >0.5 were considered for the network display using Cytoscape software^75^. The network was visualized using the edge-weighted spring-embedded layout algorithm in Cytoscape^75^. The nodes represented environmental variables and dominant bacterial and microeukaryotic OTUs and were connected by edges (significant positive or negative correlations). The resulting network had 861 edges, with 104 distinct OTUs and two environmental parameters. Six groups of edges were defined using a clustered heatmap that shows Spearman correlations previously calculated using the SparCC method. The hierarchical clustering was conducted on the transformed Spearman correlation matrix (1-M) using the hclust complete linkage method in R software.

## Electronic supplementary material

Suppl info 1

Dataset1

Dataset2
