# Supplementary material for: Deciphering biodiversity and interactions between bacteria and microeukaryotes within epilithic biofilms from the Loue River, France
Source: Sci Rep. 2017 Jun 28;7:4344. doi: 10.1038/s41598-017-04016-w (PMC5489527; doi:10.1038/s41598-017-04016-w)
Supplement: Supplementary file 1 — Suppl info 1 [file 41598_2017_4016_MOESM1_ESM.pdf]

Deciphering biodiversity and interactions between bacteria and microeukaryotes within epilithic biofilms from the Loue river, France

Anouk Zancarini[1,+], Isidora Echenique-Subiabre[1,2,+], Didier Debroas[3,4], Najwa Taïb[4], Catherine Quiblier[2,5], Jean-François Humbert[1,*]

[1] *iEES* Paris, UMR UPMC-CNRS-IRD-INRA-Univ. Paris 7-UPEC, Paris, France

[2] Unité Molécules de Communication et Adaptation des Microorganismes, Muséum National d'Histoire Naturelle, CNRS, Paris, France

[3] Laboratoire "Microorganismes: Génome et Environnement", Clermont Université, Clermont-Ferrand, France

[4] Laboratoire "Microorganismes: Génome et Environnement", CNRS, Aubière, France

[5] Université Paris Diderot, Paris, France

*jean-francois.humbert@upmc.fr

+these authors contributed equally to this work

Supplemental Table 1. Statistical analysis results of the effect of both sampling site and date on biofilm composition and diversity indices. Significance of 2-factor ANOVA tests and Tukey tests: ns, *, ** and *** indicate not significant and significant at levels of 0.05, 0.01 and 0.001 respectively.
For Tukey tests only significant results were presented in this table for both site and date effects.

		ANOVA tests			Tukey tests	
		**df**	**F value**	**P value**		**P value**
Chl-*a*	Site	3	8.9	0.0005 ***	Chamblay-Belmont	0.0007 ***
					Cleron-Belmont	0.002 **
	Date	2	3.0	0.07 ^{ns}		
	Site:Date	6	2.8	0.04 *		
Cyanobacterial biomass	Site	3	3.8	0.03 *	Parcey-Belmont	0.02 *
	Date	2	1.6	0.22 ^{ns}		
	Site:Date	6	4.5	0.004 **		
Diatom biomass	Site	3	13.0	0.00004 ***	Chamblay_Belmont	0.0007 ***
					Cleron-Belmont	0.00009 ***
					Parcey-Chamblay	0.03 *
					Parcey-Cleron	0.007 **
	Date	2	10.8	0.0005 ***	July-August	0.0004 ***
					September-August	0.04 *
	Site:Date	6	1.9	0.12 ^{ns}		
Green algae biomass	Site	3	27.0	0.0000001 ***	Chamblay-Belmont	0.000007 ***
					Cleron-Chamblay	0.0000001 ***
					Parcey-Chamblay	0.000003 ***
	Date	2	6.7	0.005 **	July-August	0.004 **
	Site:Date	6	4.0	0.007 **		
Cyanobacterial proportion	Site	3	7.8	0.0009 ***	Cleron-Belmont	0.04 *
					Parcey-Chamblay	0.007 **
					Parcey-Cleron	0.002 **
	Date	2	19.9	0.00001 ***	July-August	0.00001 ***
					September-July	0.0008 ***
	Site:Date	6	6.1	0.0007 ***		
Diatom proportion	Site	3	9.8	0.0003 ***	Cleron-Belmont	0.0007 ***
					Cleron-Chamblay	0.04 *
					Parcey-Cleron	0.0005 ***
	Date	2	34.2	0.0000002 ***	July-August	0.0000001 ***
					September-August	0.01 **
					September-July	0.0001 ***
	Site:Date	6	7.5	0.0002 ***		
Green algae proportion	Site	3	25.1	0.0000003 ***	Chamblay-Belmont	0.03 *
					Cleron-Belmont	0.0001 ***
					Parcey-Belmont	0.02 *
					Cleron-Chamblay	0.0000003 ***
					Parcey-Chamblay	0.00002 ***
	Date	2	21.0	0.000008 ***	July-August	0.000007 ***
					September-August	0.002 **
					September-July	0.05 *
	Site:Date	6	8.0	0.0001 ***		

Supplemental Table 2. Taxonomic composition of the bacterial community in the Loue River biofilms based on the normalized 16S rRNA gene sequences characterized by pyrosequencing

			Reads	Total	Number of OTU at the 97% cut-off			
**Phylum**	**Class**	**Order**	**(%)**	**seq nb**	**Total**	**Abundant**	**Interm.**	**Rare**
Acidobacteria	Acidobacteria		0.3	838	105		7	98
	Others		0.0	34	11			11
Actinobacteria	Acidimicrobiia		1.7	4911	149		30	119
	Actinobacteria		0.6	1841	179		14	165
	Thermoleophilia		0.3	932	92		6	86
	Others		0.1	209	17		2	15
Armatimonadetes			0.1	266	21		2	19
Bacteroidetes	Cytophagia	Cytophagales	0.5	1525	99		14	85
		Others	0.0	5	2			2
	Flavobacteria	Flavobacteriales	1.6	4588	106		35	71
	Sphingobacteria	Sphingobacteriales	1.1	3147	229		29	200
	Others		0.0	22	7			7
BD1-5			0.0	107	24			24
Candidate division OD1			0.1	237	50		1	49
Candidate division OP11			0.0	92	19		1	18
Candidate division SR1			0.0	41	8			8
Candidate division TM7			0.0	63	25			25
Candidate division WS3			0.0	1	1			1
Candidate division WS6			0.0	59	7		1	6
Chlamydiae			0.1	358	78		2	76
Chlorobi			0.0	51	9			9
Chloroflexi			0.3	984	145		6	139
CK-1C4-19			0.0	7	1			1
Cyanobacteria	Chroococcales		4.6	12968	67	1	18	48
	Oscillatoriales		2.6	7419	87		21	66
	Pleurocapsales		1.1	3199	22		5	17
	Others		0.0	110	16		1	15
Deinococcus-Thermus	Deinococci		1.7	4757	37		10	27
Fibrobacteres	Fibrobacteria		0.3	853	5		2	3
Firmicutes	Bacilli		1.2	3273	107		11	96
	Clostridia		1.0	2832	164		12	152
	Others		0.1	284	31		1	30
Fusobacteria			0.0	71	8		1	7
Gemmatimonadetes			0.0	103	25			25
JL-ETNP-Z39			0.0	2	1			1
Lentisphaerae	Lentisphaeria		0.0	13	6			6
Nitrospirae	Nitrospira		0.0	39	11			11
Planctomycetes	Phycisphaerae		0.1	287	56		2	54
	Planctomycetacia		5.4	15336	694		112	582
	Others		0.0	124	27			27
Proteobacteria	Alphaproteobacteria	Caulobacterales	1.2	3493	129		16	113
		Rhizobiales	9.1	25810	791		96	695
		Rhodobacterales	26.1	74420	306	7	85	214
		Rhodospirillales	0.5	1469	85		10	75
		Rickettsiales	1.0	2812	185		22	163

		Sphingomonadales	8.2	23276	349		75	274
		Others	0.1	336	38		2	36
		Unclassified	0.6	1602	96		13	83
	Betaproteobacteria	Burkholderiales	6.4	18348	327		73	254
		Methylophilales	0.2	456	8		3	5
		Rhodocyclales	0.2	486	56		5	51
		SC-I-84	0.2	486	28		3	25
		Others	0.2	602	72		9	63
		Unclassified	0.4	1151	40		7	33
	Deltaproteobacteria		0.6	1594	211		2	209
	Gammaproteobacteria	Chromatiales	0.2	504	10		3	7
		Legionellales	0.7	2001	291		10	281
		NKB5	0.2	544	98		1	97
		Pseudomonadales	0.3	846	37		6	31
		Xanthomonadales	2.5	7126	139		38	101
		Others	0.2	625	67		6	61
	Others		0.1	246	53		2	51
	Unclassified		1.5	4187	156		27	129
SM2F11			0.7	2092	44		15	29
Spirochaetes			0.0	31	3			3
Tenericutes	Mollicutes		0.1	301	24		3	21
TM6			0.1	141	47		1	46
Verrucomicrobia	Verrucomicrobiae		1.3	3690	112		22	90
	Others		0.2	438	79		3	76
WCHB1-60			0.0	22	6			6
Unclassified Bacteria			12.0	34096	843	3	81	759
Total			100	285219	7478	11	985	6482

Supplemental Table 3. Taxonomic composition of the micro-eukaryotic community in the Loue River biofilms based on the normalized 18S rRNA gene sequences characterized by pyrosequencing

**Taxonomic Affiliation**			**Reads (%)**	**Total seq nb**	**Number of OTUs at the 95% cut-off**			
					**Total**	**Abundant**	**Interm.**	**Rare**
Amoebozoa	Tubulinea	Euamoebida	3.0	838	20	1	14	5
	Unclassified		0.1	26	2		2	
Archeaplastida	Chloroplastida	Chlorophyta	68.4	18902	77	7	46	24
	Rhodophyceae	Bangiales	0.1	21	5		3	2
		Florideophycidae	0.0	1	1			1
		Unclassified	6.9	1920	12	3	6	3
	Unclassified		0.2	53	10		6	4
Excavata	Discoba	Discicristata	0.2	51	9		3	6
	Metamonada	Fornicata	0.4	110	2		2	0
		Parabasalia	0.0	2	2		1	1
Incertae sedis	Cryptophyceae	Cryptomodales	1.1	303	6		3	3
	Others		0.0	8	3		2	1
Opisthokonta	Nucletmycea	Fungi	2.6	731	73		32	41
	Others		0.2	47	14		4	10
SAR	Alveolata	Apicomplexa	7.2	1992	45	1	24	20
		Ciliophora	0.1	27	11		3	8
		Dinoflagellata	0.5	148	10		4	6
		Others	0.1	26	7		4	3
	Rhizaria	Cercozoa	2.3	634	34		18	16
		Unclassified	0.0	3	1		1	
	Stramenopiles	Chrysophyceae	0.1	28	3		2	1
		Diatomea	3.5	974	15	1	7	7
		Hyphochytriales	0.1	28	1		1	
		Peronosporomycetes	0.2	57	3		1	2
		Others	0.1	16	6		2	4
		Unclassified	0.1	27	9		5	4
Unclassified			2.5	681	75	1	28	46
Total			100	27654	456	14	223	219

Supplemental Table 4. Taxonomic composition of the microbial community in the Loue River biofilms based on the chloroplast 16S rRNA gene sequences characterized by pyrosequencing

				Reads	Total	Number of OTUs at the 97% cut-off			
**Taxonomic Affiliation**				**(%)**	**seq nb**	**Total**	**Abundant**	**Interm.**	**Rare**
Bacteria	Verrucomicrobia			0.0	2	1			1
Eukaryota	Amoebozoa	Tubulinea	Euamoebida	0.0	2	1			1
	Archeaplastida	Chloroplastida	Charophyta	1.2	306	6		6	
			Chlorophyta	2.9	749	39	1	25	13
		Rhodophyceae	Florideophycidae	0.7	184	6		5	1
			Porphyridiophyceae	0.0	4	1		1	
			Unclassified	0.2	51	1		1	
	SAR	Alveolata	Dinoflagellata	1.6	429	1	1		
		Stramenopiles	Chrysophyceae	0.0	2	1			1
			Diatomea	92.0	24027	77	17	54	6
			Xanthophyceae	1.1	289	8		8	
Unclassified				0.3	79	4		4	
Total				100	26124	146	19	104	23

Supplemental Table 5. Permanova analyses of the effect of both sampling site, date and their interaction on microbial communities composition within biofilms (16S rRNA bacterial, 18S rRNA micro-eukaryotic and 16S rRNA chloroplast data sets). Significance of Permanova tests: ns, *, ** and *** indicate not significant and significant at levels of 0.05, 0.01 and 0.001 respectively

		df	F value	R^2	P value
16S rRNA bacterial	Site	3	7.2	0.33	0.0001 ***
	Date	3	4.3	0.20	0.0001 ***
	Site:Date	4	2.3	0.14	0.0003 ***
	Residuals	22		0.33	
18S rRNA micro-eukaryotic	Site	3	9.5	0.25	0.0001 ***
	Date	3	17.3	0.45	0.0001 ***
	Site:Date	4	3.4	0.12	0.0005 ***
	Residuals	22		0.19	
16S rRNA chloroplastic	Site	3	9.7	0.37	0.0001 ***
	Date	3	4.6	0.18	0.0001 ***
	Site:Date	4	3.2	0.17	0.0001 ***
	Residuals	22		0.28	

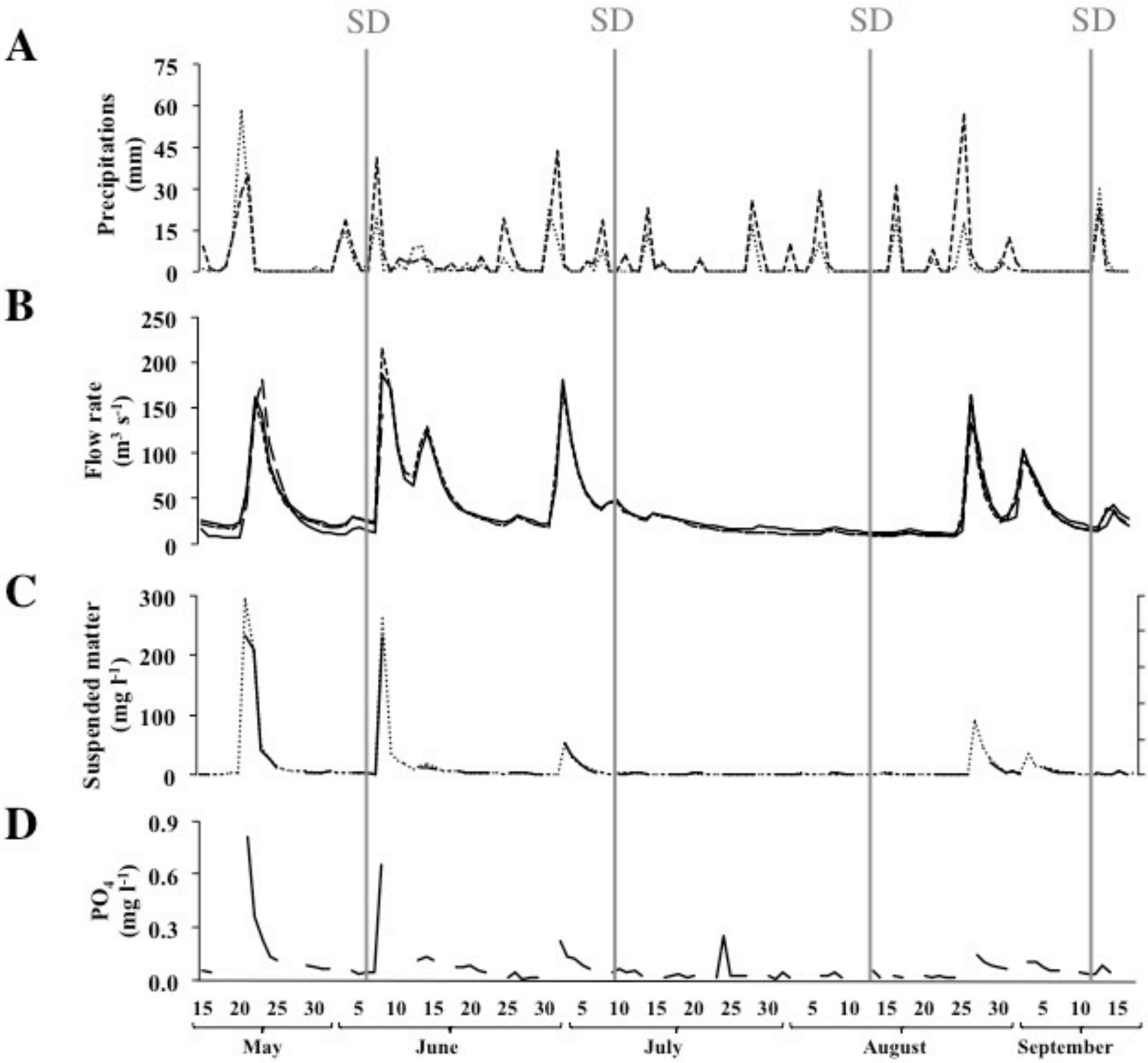

Supplemental Figure 1. (A-B) Environmental parameters measured during the sampling period at different stations on the Loue River (Chenecey-Buillon station = small dash, Champagne-sur-Loue station = solid line, Parcey station = long dash and Dole-Tavaux station = dots); (C-D) Physico-chemical parameters at Chenecey station (suspended matter and phosphates = solid lines; turbidity = dots). SD = Sampling date during our study.

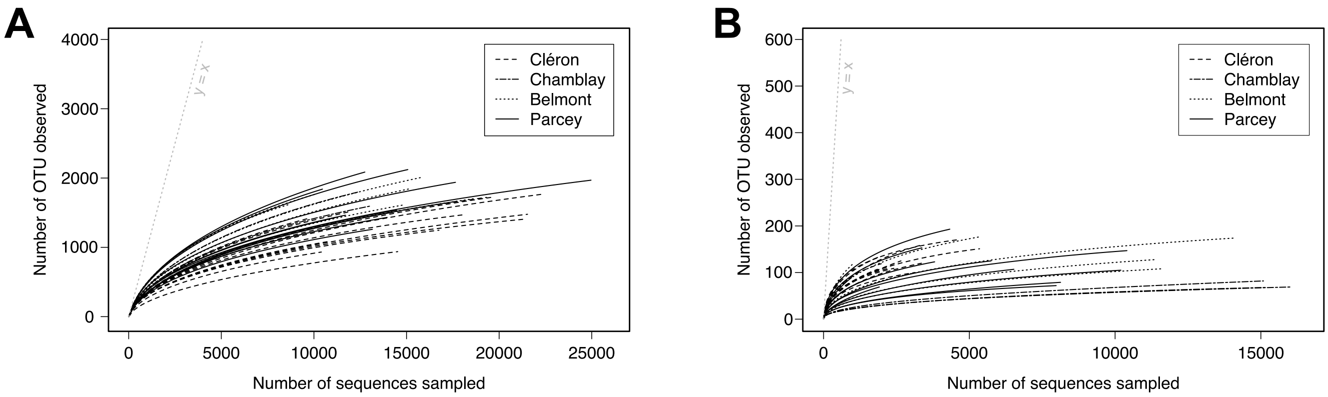

Supplemental Figure 2. Rarefaction curves for (A) bacterial (16S rRNA gene sequences without chloroplast sequences) and (B) micro-eukaryotic (18S rRNA gene sequences) communities. Rarefaction curves were calculated using the vegan package. The three replicates from each site and date are represented. Samples from: Cléron = dash, Chamblay = dash/dot, Belmont = dot and Parcey = continuous line.

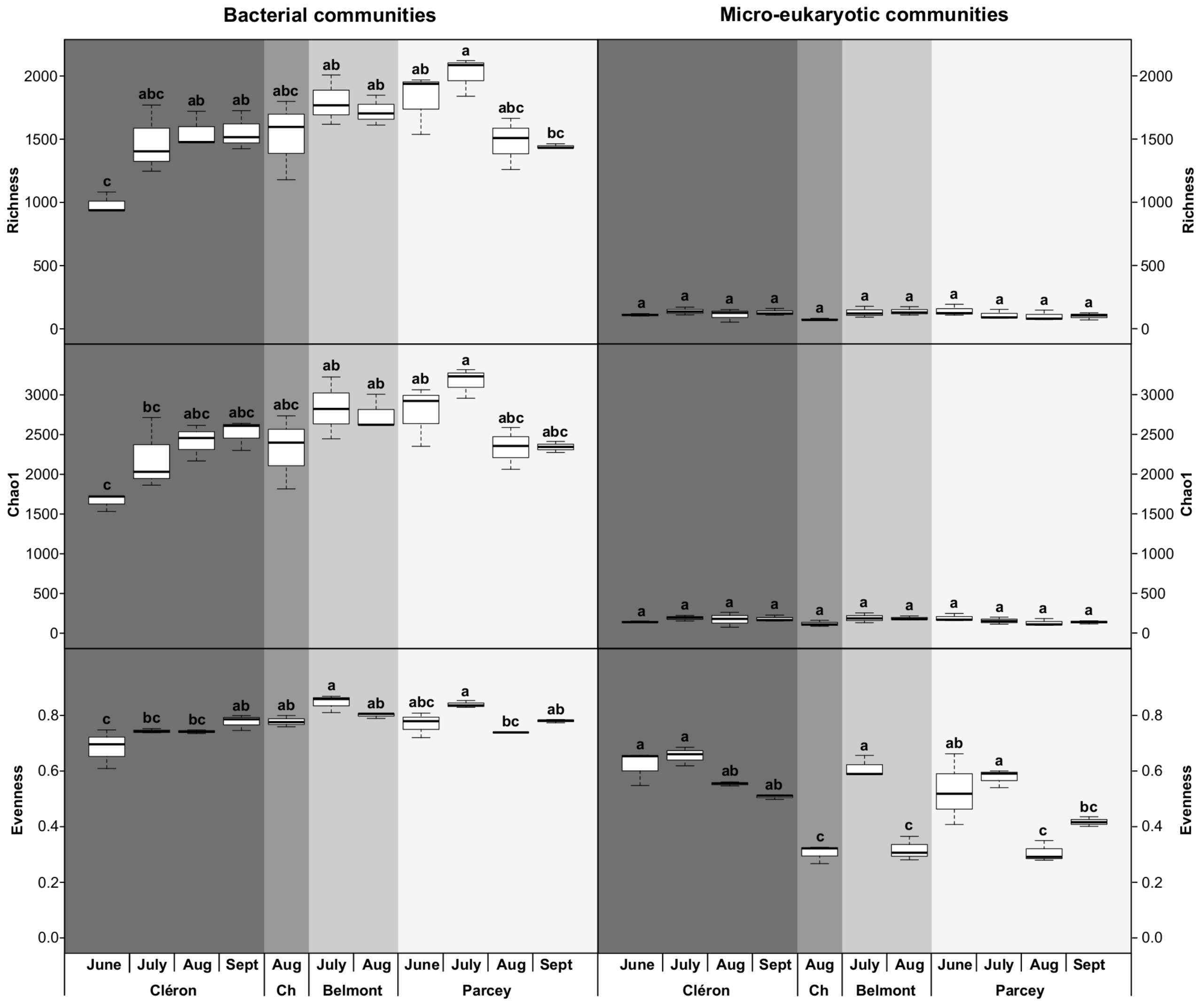

Supplemental Figure 3. Diversity indices in the bacterial (16S rRNA gene sequences without chloroplast sequences) and micro-eukaryotic (18S rRNA gene sequences) communities. Letters with different labels indicate significant differences ($p < 0.05$). Richness and evenness represent the 97% (Bacteria) and 95% (Micro-eukaryotes) OTU number and Pielou's evenness, respectively. Abbreviations: Aug, August; Sept, September; Ch, Chamblay.

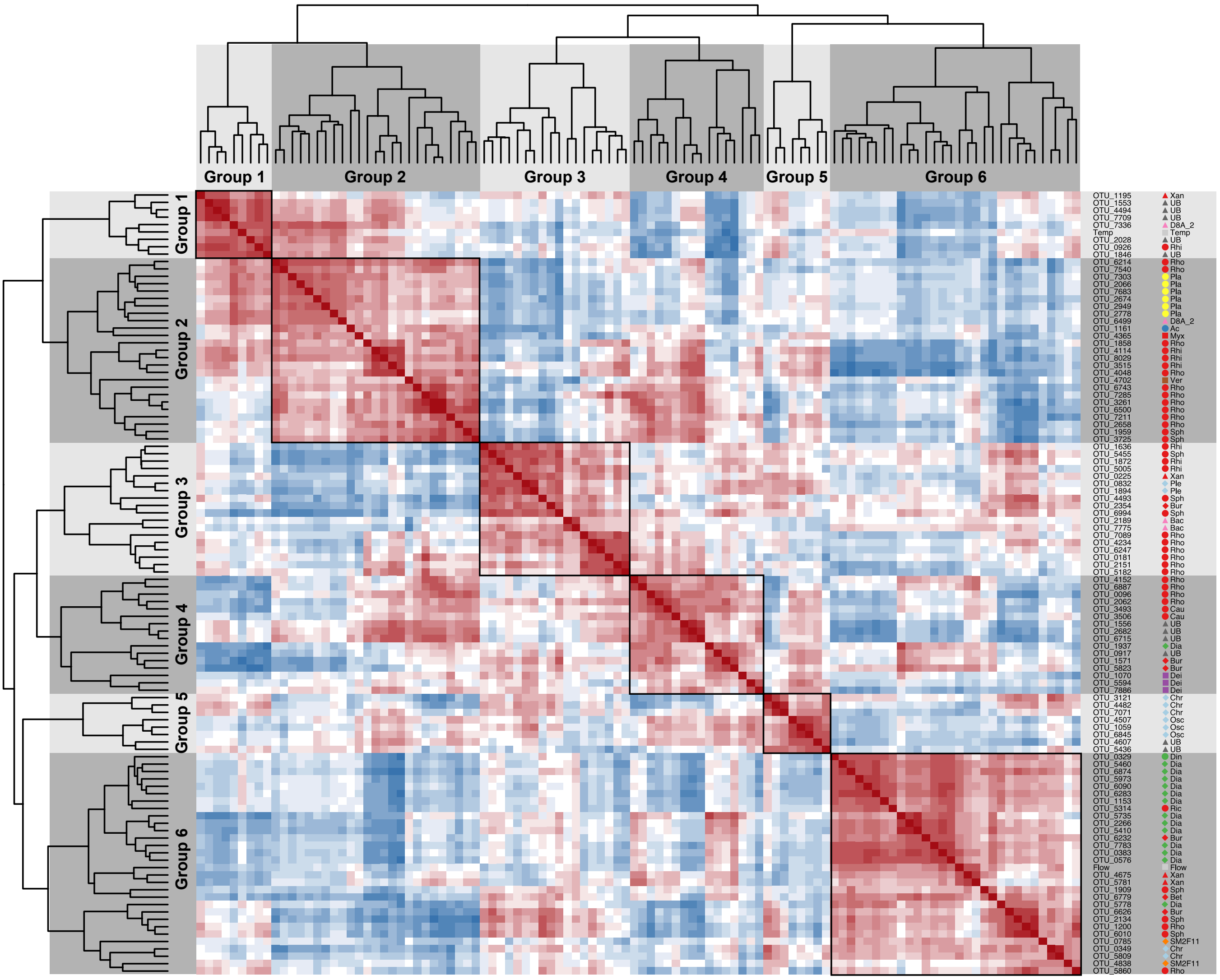

Supplemental Figure 4. Associations among dominant microbial OTUs (≥1% of the total sequences for at least one of the 33 samples) and environmental variables (water temperature and Loue river flow). This clustered heatmap shows Spearman correlations calculated using the SparCC method (Sparse Correlations for Compositional data72; available at https://bitbucket.org/yonatanf/sparcc) among relative abundance levels of dominant bacterial and micro-eukaryotic OTUs (based on the 454 pyrosequencing data set of the 16S bacterial and chloroplast rRNA gene sequences) and environmental data for all of the samples (n = 33). Hierarchical clustering was generated using hclust complete linkage method in R. Red and blue colors indicate positive and negative correlations respectively. Color intensity indicates the degree of correlations evaluated by coefficient of correlations (more intensive color indicates higher coefficient).
